# Proteolysis in *Helicobacter pylori*-Induced Gastric Cancer

**DOI:** 10.3390/toxins9040134

**Published:** 2017-04-11

**Authors:** Gernot Posselt, Jean E. Crabtree, Silja Wessler

**Affiliations:** 1 Division of Microbiology, Department of Molecular Biology, Paris-Lodron University of Salzburg, Salzburg 5020, Austria; gernot.posselt@sbg.ac.at; 2 Leeds Institute Biomedical and Clinical Sciences, St. James’s University Hospital, University of Leeds, Leeds LS9 7TF, UK; j.crabtree@leeds.ac.uk

**Keywords:** *Helicobacter pylori*, protease, MMP, ADAM, TIMP, E-cadherin, HtrA, EGFR

## Abstract

Persistent infections with the human pathogen and class-I carcinogen *Helicobacter pylori* (*H. pylori*) are closely associated with the development of acute and chronic gastritis, ulceration, gastric adenocarcinoma and lymphoma of the mucosa-associated lymphoid tissue (MALT) system. Disruption and depolarization of the epithelium is a hallmark of *H. pylori*-associated disorders and requires extensive modulation of epithelial cell surface structures. Hence, the complex network of controlled proteolysis which facilitates tissue homeostasis in healthy individuals is deregulated and crucially contributes to the induction and progression of gastric cancer through processing of extracellular matrix (ECM) proteins, cell surface receptors, membrane-bound cytokines, and lateral adhesion molecules. Here, we summarize the recent reports on mechanisms how *H. pylori* utilizes a variety of extracellular proteases, involving the proteases Hp0169 and high temperature requirement A (HtrA) of bacterial origin, and host matrix-metalloproteinases (MMPs), a disintegrin and metalloproteinases (ADAMs) and tissue inhibitors of metalloproteinases (TIMPs). *H. pylori*-regulated proteases represent predictive biomarkers and attractive targets for therapeutic interventions in gastric cancer.

## 1. Introduction: The Gastric Epithelium in Health and Disease

The gastric mucosa is in perpetual contact with ingested nutrients, chemicals and pathogens and thus needs to form a protective layer as a first line of gastrointestinal defense. The epithelial barrier function is provided by a tight network of different cell types with numerous gastric glands releasing gastric acid or hormones and goblet cells that produce a protective mucus layer covering gastric epithelial cells [1]. A loose connective tissue, the lamina propria mucosae, and sheet of myofibroblasts build the lower layers of the gastric lining [2,3]. The barrier function requires an intact architecture of the epithelium. To maintain the epithelial structures, cells are interconnected by several types of intercellular junctions, including tight junctions (TJ) and adherens junctions (AJ). TJs are located between apical and basolateral cell surface domains and prevent free passage of solutes over the epithelium. Intact TJs are critical in maintaining a polarized epithelium [4]. Functionally, TJs are comprised of transmembrane proteins like claudins, occludins and JAMs and intracellular scaffolding proteins like the zonula occludens proteins (ZO1-3), cingulin, afadin and others that bridge the intercellular junctions to the actin cytoskeleton and microtubules [5]. Directly below, epithelial cadherins (E-cadherins) constitute homotypic interactions and form AJs. AJ are composed of membrane spanning cadherins which are linked to the cytoskeleton via a catenin complex including α-catenin, β-catenin and p120. E-cadherin was shown to be crucially involved in embryogenesis as well as in tissue homeostasis and is considered as an important tumor suppressor. In healthy tissues, loss of cell-cell contact is sensed via disruption of intercellular E-cadherin interaction and via the β-catenin—WNT signaling axis proliferation is stimulated in order to close epithelial micro-lesions. However, loss of E-cadherin and aberrant c-Met activation is also a hallmark in in epithelial—mesenchymal transition (EMT) in the tumorigenesis of gastric cancer [6,7,8,9].

### Helicobacter Pylori-Dependent Gastric Pathologies 

The bacterial pathogen *Helicobacter pylori* (*H. pylori*) has been classified as a class-I carcinogen [10] since it was identified as the leading cause for inflammation-driven gastric cancer [10]. Normally, infections with *H. pylori* are acquired in early childhood and persist lifelong if not treated by antibiotics. The majority of infected persons do not develop clinically relevant symptoms; however, a significant number of patients develop chronic gastritis, ulceration of the stomach and duodenum, MALT lymphoma, and gastric cancer. The reason why some individuals develop severe disease is still not fully clear, but depends on the interplay between bacterial virulence factors and genetic predispositions of the host. In this context, genes encoding interleukins (e.g., IL-1β), tumor necrosis factor alpha (TNF-α), cyclooxygenase-2 (COX2), and other host factors have been associated with an elevated risk for gastric cancer [11,12]. Among a wide range of virulence factors the bacterial effector protein cytotoxin-associated gene A (CagA) attracted much attention, which is an intensively investigated bacterial effector protein. CagA is translocated into the host cytoplasm via a specialized type IV secretion system encoded by the *cag* pathogenicity island (*cag*PAI). After translocation, CagA is tyrosine-phosphorylated by Src kinases and c-Abl [13,14]. Non-phosphorylated and tyrosine-phosphorylated CagA can deregulate certain signal transduction pathways leading to depolarization of the epithelium [14,15]. The vacuolating cytotoxin A (VacA), adhesins such as blood-group-antigen-binding adhesion (BabA), sialic acid binding adhesin (SabA), adherence-associated lipoprotein A and B (AlpA/B), and outer inflammatory protein A (OipA) or the serine protease high temperature requirement A (HtrA) are additional examples of described bacterial factors which can directly interfere with host cells or the extracellular matrix (ECM) to manipulate the epithelial barrier and have been summarized in recent review articles [15,16,17].

Chronic inflammation and neoplastic tissue transformation are accompanied by *H. pylori*-mediated disruption of the inherent gastric epithelium. This requires a network of complex interspecies mechanisms of which *H. pylori*-controlled proteolysis represents a central and manifold step. Though many proteases, including the metzincin proteases, are active in healthy tissues and play crucially important roles in tissue homeostasis and remodeling, overshooting expression and aberrant activation are hallmarks of chronic inflammation and tissue transformation. In *H. pylori* infections, several proteolytic cascades have been described and bacterial as well as host proteases participate in deregulating the ECM and healthy tissues. In fact, the influence of chronic *H. pylori* infections on the expression of host proteases is highly complex and many intracellular, secreted or membrane attached proteolytic cascades are affected. The possible functions of intracellular proteases in gastric cancer have been summarized in several excellent reviews [18,19,20] and involve inflammatory [21,22] and apoptotic caspases [23,24], altered proteasomal targeting and degradation [25,26,27], but also proteases like calpains [28] or cathepsins [29]. Therefore, in this review we focus on *H. pylori*-secreted and *H. pylori*-upregulated epithelial extracellular membrane-anchored and secreted proteases and include members of a disintegrin and metalloproteinase (ADAMs) family, the matrix metalloproteinases (MMPs) (Table 1), and their regulatory counterparts—the tissue inhibitors of metalloproteinases (TIMPs), which concertedly have drastic consequences on the architecture of the *H. pylori*-colonized gastric epithelium. Their primary functions, the general substrates and the phenotype of knockout animals have been summarized in a number of excellent reviews [30,31,32,33,34]. 

## 2. Deregulation of Extracellular Host Proteases by *H. pylori*

### 2.1. A disintegrin and Metallopeptidase ADAM

**Figure 1:**
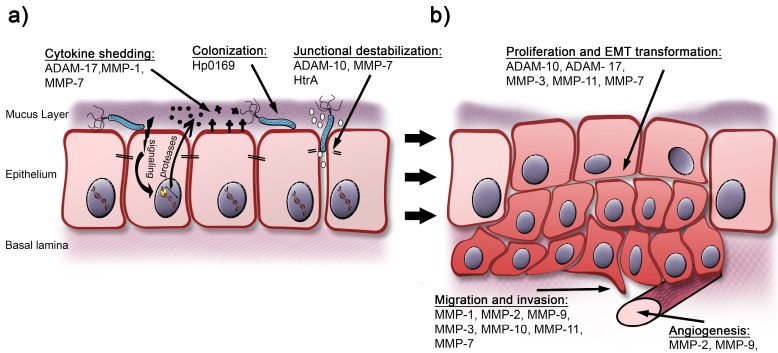
Model of *H. pylori*-regulated and secreted proteases in early and late pathogenesis. (**a**) *H. pylori* induces the transcription of a wide range of host ADAM and MMP proteases or secretes bacterial proteolytic activities, which can directly shed cytokines, interfere with ECM proteins or lateral junction complexes; (**b**) In advanced stages of *H. pylori* pathogenesis, proteases are implicated in proliferation and EMT processes, but also in tumor cell migration, invasive growth and angiogenesis. For more details, see text.

The ADAM proteases are zinc-dependent, membrane-anchored multidomain proteins that contain a prodomain, the metalloproteinase domain, a disintegrin domain, a cysteine-rich domain, an EGF-like domain, a transmembrane domain, and a cytoplasmic domain. Their functional repertoire covers processes as diverse as migration, adhesion, signalling, and of course proteolysis. The proteolytic targets of the ADAM family span ECM proteins and adhesion molecules but also cytokines and growth factors and their cognate receptors [35]. Amongst others, ADAM-10 and -17 are particularly important during development as reflected by the early embryonic lethality in knock-out mice. However they also have a well-established role in chronic inflammation and tumorigenesis, especially migration and proliferation in metastatic tumors [31,33]. ADAM expression has been described to be upregulated in individuals with *H. pylori* gastritis (ADAM-10, -17, -19) and gastric cancer (ADAM-9, -10, -12, -15, -17, -19, -20) [36,37,38]. Among those, ADAM-10 expression was more frequent in patients infected with *cag*PAI-negative strains [39]. The elevated tissue expression is supposed to be a combination of elevated expression in the gastric epithelial cells and an infiltrate of activated immune cell that inherently express high levels of ADAM proteins [31]. ADAM-17 was shown to be activated in a CagL-dependent manner [40], but in other reports, stimulation of ADAM-17 in epithelial cells was independent of a functional *cag*PAI [41] or occurred in response to lipopolysaccharide (LPS) [42] (Table 2, Figure 1). Once activated ADAM-17 contributes to TNF-α, TGF-α (transforming growth factor alpha) and importantly to HB-EGF (heparin-binding EGF-like growth factor) shedding [41,42]. Downstream EGFR (epidermal growth factor receptor) activation was shown to be strictly dependent on the c-terminus of ADAM-17 [41] and could be inhibited by abrogation of interleukin-8 (IL-8) signaling [43] or the Rac1-p38 signaling axis [42]. The ADAM-17-mediated activation of EGFR signaling is of particular interest as it was shown to prevent gastric epithelial apoptosis [44] and for its importance in the formation of pre-neoplastic lesions in the gastric mucosa as demonstrated in a gerbil model [45]. *H. pylori* also activates ADAM-10 [46,47], which was shown to contribute to shedding of the AJ molecule E-cadherin and the receptor tyrosine kinase c-Met in *H. pylori*-infected NCI-N87 cells [47]. A recent publication shows that ADAM-10 and -17 mediated shedding of Notch1 contributes to the stem-like phenotype of gastric cancer stem cells and their inhibition reduces the capacity of anchorage independent proliferation [48] (Table 2, Figure 1) suggesting that *H. pylori*-activated proteases of the ADAM family could exert direct effects on neoplastic transformation.

### 2.2. Matrix Metalloproteinases MMP

Akin to ADAM proteases, MMPs belong to the family of zinc dependent metzincin proteases. In humans MMPs are encoded by approximately 24 genes which encode soluble and membrane anchored proteases. Most MMPs are produced in a latent version where the protease is kept inactive via a “cysteine switch” in the prodomain. Permanent activation of MMPs is facilitated via proteolytic removal of the prodomain, whilst tissue inhibitors of metalloproteinases (TIMPs) serve as endogenous inhibitory proteins. MMPs are commonly classified as membrane attached MMPs or soluble MMPs, the latter group is—according to their substrate specificity—subdivided into collagenases, gelatinases, stromelysins and a fourth group with more diverse substrates (Table 1) [32]. MMPs were initially identified as proteases responsible for ECM turnover. However, it is now evident that MMPs play important roles in many gastrointestinal pathologies, including chronic infections and cancer. Moreover, MMPs were also described to contribute to E-cadherin cleavage and support EMT [49]. Unsurprisingly regulation of the MMP protease family has also been associated with *H. pylori*-dependent diseases. Different MMPs are upregulated in the *H. pylori-*infected gastric mucosa and gastric cancer. Like the ADAMs, MMPs are produced at higher levels by gastric epithelial cells as well as by infiltrating lymphocytes and macrophages. In the gastric adenocarcinoma cell line AGS enhanced gene expression after *H. pylori* infection was observed for MMP-1, -3, -7, -8, and -10 [50,51] whereas increased expression of MMP-9 after *H. pylori* infection was only seen in MKN28 and MKN45 cell lines [52]. In biopsies of *H. pylori*-associated gastritis and gastric cancer MMP-1, -2, -7, -8, -9, -10, -11, -12, and MMP-14 were upregulated [50,53,54,55,56,57] (Table 2, Figure 1). 

#### 2.2.1. *H. pylori*-Induced MMPs: The Collagenases MMP-1, MMP-8, and MMP-13

Among MMPs, a subgroup of collagenases (MMP-1, -8, -13) containing a hemopexin-like domain can cleave triple-helical collagens [32]. MMP-1 degrades collagen and other ECM proteins including vitronectin and fibronectin, but also cleaves cytokines like proTNF-α and proIL-1β. Furthermore, it was shown to process and activate proMMP-2 and proMMP-9 [58] and is associated with poor prognosis in cancer [59]. In *H. pylori* infections, MMP-1 upregulation was reported [60] and suggested to depend on the *cag*PAI [50] and the outer membrane protein OipA [61]. The activation of MMP-1 expression was described to employ signalling cascades involving protein kinase C (PKC), c-Jun N-terminal kinases (JNK), extracellular-signal regulated kinase (Erk) and p38 [50,60,61,62,63]. In healthy individuals gastrin was suggested as a factor regulating MMP-1 expression, again in a PKC and Erk dependent fashion [64]. In in vitro invasion assays *H. pylori* induced migration across a collagen matrix was critically dependent on MMP-1 activity [62] which underlines its importance in metastasis of gastric cancer. MMP-8 was shown to be upregulated in gastric epithelial cells and elevated levels of MMP-8 could be observed in sera from *H. pylori* associated gastritis patients [50,55]; however little is known about its regulation and its biological relevance. Collagenase 3 (MMP-13) was shown to be upregulated in murine *H. felis* infection models [65] however, it is induced less than two-fold in MKN-1 cells [66]. The experiments by Sokolova et al. suggest that the MMP-8 and -13 do not suffice for invasive migration [62] (Table 2, Figure 1). Still, the in vivo contribution of the individual MMP collagenases to *H. pylori-*associated disease has been hardly addressed and requires future attention. 

#### 2.2.2. *H. pylori*-Induced MMPs: The Gelatinases MMP-2, and MMP-9

In contrast to collagenases, gelatinases degrade gelatin or non-helical regions of collagen. Both gelatinases, MMP-2 and MMP-9, contribute to ECM turnover and are particularly critical for targeting type IV collagen in the basement membrane. Furthermore, both gelatinases were shown to be important for IL-1β dependent recruitment of neutrophils via processing of the chemokine CXCL5 [67]. They are thought to contribute to inflammation, tissue remodeling and tumor metastasis and were suggested as prognostic markers in serum and plasma of gastric cancer patients [68,69,70,71]. The expression of MMP-2 and MMP-9 is significantly higher in gastric biopsies obtained from *H. pylori* infected individuals as compared to healthy controls. For MMP-9 it was shown, that elevated expression is linked to elevated numbers and higher expression levels in tissue infiltrating macrophages, which also produce MMP-9 upon *H. pylori* infection in vitro [72], and expression decreases significantly after successful *H. pylori* eradication therapy [73]. MMP-9 expression was attributed to CagA phosphorylation in AGS cells in an Erk and NF-κB (nuclear factor kappa B) dependent fashion; however, in murine infection models employing CagA-negative *H. pylori* or *H. felis* strains no CagA dependency could be established [74,75,76]. Furthermore, the Th-17 associated cytokine IL-21 was suggested to promote MMP-2 and MMP-9 production in the gastric cancer cell lines AGS and MKN-28 independent of MAPK activation [77]. In conclusion, the gelatinases MMP-2 and in particular MMP-9 have been linked to cancer progression in several instances including invasive growth, metastasis and tumor associated angiogenesis [32,78] (Table 2, Figure 1).

#### 2.2.3. *H. pylori*-Induced MMPs: The Stromelysins MMP-3, MMP-10, and MMP-11

All stromelysins (MMP-3, -10, and -11) have been reported to be upregulated in *H. pylori* infected gastric epithelial cells or *H. pylori* associated gastric cancer [50,56,57,60,79]. MMP-3 was shown to promote EMT and was suggested to be a natural tumor promoting factor [80,81]. MMP-3 expression in response to *H. pylori* was suggested to depend on the presence of phosphorylated CagA EPIYA motifs in gastric adenocarcinoma cell lines and is regulated alike the EMT markers ZEB1 (zinc finger E-box-binding homeobox 1), vimentin, snail, and CD44 [82]; However, this dependency on CagA was not observed in mouse infection models [75]. It is conceivable, that the different regulation in vivo might be attributed to IL-1β dependent secretion of MMP-3 [79]. MMP-3 levels increase with tumor progression [83] and elevated serum levels provide, in combination with MMP-7, a marker for poor prognosis in gastric cancer [84]. Functionally, MMP-3 was linked to cell migration in invasion assays with MMP-3 silenced AGS cells [85], even MMP-3 derived from tumor-associated myofibroblasts was sufficient to promote AGS cell migration [86]. MMP-10 was shown to be upregulated in *H. pylori* infected gastric cancer lines in an EGFR-, Src-, and Erk-dependent manner. Though not entirely dependent on CagA injection or phosphorylation, the highest levels of MMP-10 were observed upon infection with CagA-translocating strains [51]. MMP-10 expression was also reported in *H. pylori* positive gastric biopsies [51] and expression of MMP-10 increases with progression of cancer to later stages [56]. Moreover, MMP-10 was shown to promote invasive growth as silencing led to reduced transmigration of AGS in matrigel-coated chambers [51]. Similarly, MMP-10 from tumor associated macrophages was shown to increase AGS cell migration [87]. MMP-11 is also expressed in gastric cancer and expression levels increase with cancer progression. Furthermore, expression levels correlate with higher expression of IGF-1 (insulin-like growth factor 1) which is described as proliferation—and migration promoting factor [57]. Finally, silencing of MMP-11 in gastric adenocarcinoma cells leads to a reduction in IGF-1 production with concomitant reduced proliferation and invasive migration [88] (Table 2, Figure 1). Given the fact, that silencing of each stromelysin-type MMP is sufficient to abrogate cell invasion it is likely, that all three are necessary and regulate cell migration in a cooperative manner.

#### 2.2.4. Matrilysin, Macrophage Metallo-Elastase, and Membrane-Type MMPs

MMP-7 (matrilysin) lacks the hemopexin-like domain and cannot cleave intact collagen. Still, it targets a wide array of ECM proteins but was also suggested as a sheddase for TNF-α, HB-EGF or E-cadherin. It was shown to be crucially involved in gastrointestinal defense and epithelial wound healing. However, MMP-7 was repetitively shown to contribute to invasive growth and enhanced proliferation in malignant cells [32]. MMP-7 expression is higher in *H. pylori* associated gastritis and upregulation is demonstrated in cell culture experiments [89,90,91]. Expression is dependent on the presence of an intact *cag*PAI; however, no influence of CagA could be established [90,91,92,93]. Its expression is mediated via NF-κB and MEK (mitogen-activated protein/ERK kinase) and requires p120 mediated liberation from Kaiso repression [89,94]. MMP-7 regulation was previously associated with gastrin levels [64,95] and it was suggested, that also *H. pylori* induced MMP-7 production is critically dependent on gastrin [93]. MMP-7 silencing results in lower levels of HB-EGF shedding and simultaneously in reduced expression of EMT markers [93], whereas antibody neutralization of MMP-7 reduced the migratory and invasive potential in AGS cells [89]. Furthermore, MMP-7 was found in metaplastic lesions in gerbil infection models [96], which supports the observation that it releases the growth factor IGFII from myofibroblasts by cleaving IGFBP5 (insulin like growth factor binding protein 5) [97]. This mechanism however seems to be not only disease promoting, but also serves the sensing of epithelial lesions and wound healing. This is supported by the observation, that MMP-7 knockout mice suffer from exacerbated inflammation upon *H. pylori* infection and MMP-7 could also play a protective role in vivo [98]. Though MMP-7 increases drastically in gastric cancer, it is of low prognostic value as marker, unless combined with MMP-3 [71,84]. MMP-12 (macrophage metalloelastase) is required for macrophage transmigration trough the basement membrane and appears to be the physiologic activator of angiostatin which counteracts neoangiogenesis [32] (Table 2, Figure 1). MMP-12 is not detectable in *H. pylori* infected gastric cancer cell lines; however its presence was shown in gastric cancer tissue. Little is known about its regulation and function in *H. pylori*-associated disease. Though contradictory reports exist concerning its prognostic value, it was suggested that MMP-12 expression indeed is beneficial and correlates with higher survival rates amongst gastric cancer patients [99,100]. Of the membrane type MMPs only MMP-14 (MT1-MMP) was reported to be upregulated in *H. pylori* gastritis; however, little is known about its role in disease [53].

### 2.3. Tissue Inhibitors of Metalloproteinases TIMPs

Tissue homeostasis critically depends on ECM remodeling and controlled expression and regulation of the contributing proteases. The regulation of proteolytic activities is facilitated at several levels and includes also inhibitory binding partners, the TIMPs. In vertebrates four different TIMPs (TIMP-1-TIMP-4) are expressed which bind to and inhibit proteases in a 1:1 ratio. Thus not only the expression levels of ADAMs and MMPs are of interest, but also the ratio between proteases and their inhibitors. Unsurprisingly, knockout animals show phenotypes which can mostly be attributed to excessive protease activity [31]. However, in recent years it became evident, that TIMPs also have direct cytokine functions and are able to mediate signalling independent of their protease inhibitory capacity. This influences several processes related to cell growth, apoptosis, cell differentiation or angiogenesis [101,102]. The balance between the protease- and the cytokine activities of TIMP-1 in cancer was also associated with its glycosylation pattern [103]. TIMP-1-TIMP-3 are able to inhibit all active MMPs whilst TIMP-4 shows a more selective pattern of inhibition. Furthermore, TIMP-1 and TIMP-4 have been shown to bind proMMP-9 whereas TIMP-2 binds proMMP-2. TIMP-3 was shown to bind both pro-gelatinases [101]. Moreover, TIMP-1, -3, and -4 act as negative regulators of ADAM-10 and in particular of ADAM-17 [34]. In vivo, TIMP-3 was suggested as the major player in MMP inhibition; furthermore it is critically important for controlling ADAM-17 mediated TNF-α release [34]. In tissue extracts from *H. pylori* gastritis TIMP-1 and TIMP-2 protein levels are not elevated as compared to healthy controls. However, TIMP-1 was observed to be attached to MMPs in infected individuals, whereas the free protein was only detectable in *H. pylori* negative biopsies [72]. The association with MMPs could also help to explain the reduced serum levels of TIMP-1 in *H. pylori* gastritis [55]. Others found TIMP-1, -3, and -4 expressed at higher levels in the corpus of *H. pylori* infected stomachs [104]. The upregulation of TIMP-3 was also shown in gastric epithelial cells in vitro [60] where it was shown to form complexes with MMP-3 [79]. A higher level of TIMP-1 expression is seen in gastric ulcers where it was suggested as a distinctive marker for discriminating *H. pylori* and NSAID related ulcers [105,106]. All TIMPs were found to be deregulated in gastric cancer [54,71,83] and polymorphisms in the loci of TIMP-1 and TIMP-2 have been associated with intestinal metaplasia or poor prognosis, respectively [107,108]. The prominent role of TIMP-3 in regulating proteolysis in vivo is highlighted by the observation, that expression levels of TIMP-3 decline while MMP-3 levels increasing during progression of gastric cancer [83] (Table 2, Figure 1). This shifts the equilibrium towards the protease and thus facilitates the detrimental effects of overshooting proteolysis. 

## 3. *H. pylori* Expresses Proteases Exhibiting a Direct Influence on the Integrity of the Epithelial Barrier Function

Besides controlled proteolysis in cell adhesion or signal transduction via regulated host proteases, *H. pylori* also expresses and secretes bacterial proteases, which can directly interfere with tumor-suppressive functions of the ECM and E-cadherin. Generally, the knowledge about *H. pylori*-secreted proteases is still limited, but in the last years numerous studies were reported indicating that *H. pylori* expresses several proteases exhibiting direct effects on the infection process. One of the first reports described a <50 kDa protease of *H. pylori* that degrades the protective mucus layer leading to an increased cell injury by the gastric acid [109]. The protease was not identified at this time, but possibly the same shed *H. pylori* protease degrades platelet-derived growth factor (PDGF) and transforming growth factor beta (TGF-beta), which could be blocked by sulglytide [110,111]. Using an antibody against elastase of *Pseudomonas aeruginosa*, haemagglutinin/protease (HAP) was detected in *H. pylori* supernatants that exhibited mucinase activity [112]. A few years later, another bacterium-derived protease activity in the supernatants of *H. pylori* was detected with a molecular weight of approximately 200 kDa [113]. Although this enzyme was also not identified, it was further characterized as a zinc-dependent and calcium-stabilized metalloprotease. Specific substrates were not analyzed, but the authors hypothesized that the extracellular localized protease could exert possible functions in the pathogenesis of *H. pylori* [113]. 

A first functional study on a *H. pylori* protease was published in 2003 [114]. In *H. pylori*-infected Mongolian gerbil, the secreted calcium-dependent collagenase Hp0169 was identified as an important factor for efficient colonization in vivo [114]. Interestingly, recombinant Hp0169 protein degrades native type-I collagen indicating that Hp0169 targets helical collagen during infection; a fact that differentiates Hp0169 from prevalent gelatinases. Hp0169 knock-out mutants failed to colonize gerbil stomachs [114] suggesting that Hp0169-mediated type-I collagen degradation facilitates bacterial adherence (Table 2, Figure 1). Whether all *H. pylori* and possibly non-*pylori Helicobacter* isolates express Hp0169 and whether its expression correlates with *H. pylori*-dependent gastric cancer has not been investigated to date. 

Based on the observation that *H. pylori* can secrete proteolytically active proteases that can directly interfere with the epithelium, a comprehensive screen for additional genes encoding “putative” or “hypothetical” proteases exhibiting an extracellular localization was performed. Among several predicted candidate genes, the serine protease and chaperone high temperature requirement A (HtrA) was identified as an interesting caseinolytic active enzyme [115]. HtrA proteins are widely expressed by bacteria. In a comprehensive characterization of worldwide *H. pylori* strains, HtrA-negative isolates were not found [116]. Supporting the idea that HtrA is expressed in non-*pylori Helicobacter* strains as well, HtrA expression was also observed in Bengal tiger isolates [117]. HtrA was previously described as a secreted protease found in the supernatants of *H. pylori* [118,119], while possible biological functions in *H. pylori* pathogenesis were unknown. Generally, HtrA proteases exhibit important chaperone functions in bacteria through refolding and degradation of misfolded proteins; therefore facilitating bacterial growth and survival under stress conditions [120,121,122]. Correspondingly, *H. pylori* HtrA is highly stable and active under extreme conditions such as varying temperatures, pH or salt concentrations [123]. The question whether these characteristics contribute to virulence in the hostile environment of the human stomach is still unanswered. The main reason for this lack of knowledge is the unavailability of an *htrA*-negative *H. pylori* isolate or a genetically generated *htrA* knock-out mutant [116] indicating that HtrA expression is essential for *H. pylori* physiology [124]. Therefore, it was assumed that HtrA can facilitate *H. pylori* colonization of the stomach through improving bacterial fitness.

### H. pylori HtrA Induced E-Cadherin Ectodomain Shedding

An additional aspect in HtrA functions was added by the finding that the proteolytic activity of *H. pylori* HtrA also exhibits direct effects on the infection process. Extracellular HtrA cleaves the cell adhesion protein and tumor suppressor E-cadherin of gastric epithelial cells [46,125]. The extracellular matrix protein fibronectin was identified as an additional substrate for *H. pylori* HtrA [46] (Table 2, Figure 1). In fact, the finding that fibronectin is a substrate for *H. pylori* HtrA is interesting and leads to the question whether cleavage of fibronectin facilitates the translocation of the oncoprotein CagA? It has been previously shown that the type-IV secretion system-mediated CagA translocation into the cytoplasm of infected epithelia cells requires binding of CagL and additional components encoded by the cag pathogenicity island to α5β1 integrins [126,127]. It is not completely clear if the RGD motif in the CagL proteins competes with the binding of the RGD motif containing fibronectin molecule to β1 integrin [126,127], but HtrA-mediated degradation of fibronectin might enhance the accessibility of β1 integrin for *H. pylori* to increase the translocation of the CagA oncoprotein and subsequently cancer-associated signal transduction.

Identification of E-cadherin as an HtrA substrate further underlines the significance of HtrA activity in *H. pylori*-induced carcinogenesis. E-cadherin is an important key molecule in the epithelial architecture and establishes functional intercellular adhesions. It is enticing to surmise that HtrA-mediated E-cadherin shedding can affect the gastric epithelial integrity to promote *H. pylori* infections. *H. pylori*-induced disruption of adherens junctions via E-cadherin shedding opens the intercellular space for the bacteria allowing transmigration across the epithelium [46]. In fact, intercellular *H. pylori* was observed in biopsies of gastric cancer patients [128] (Table 2, Figure 1). 

E-cadherin also functions as a tumor suppressor and dysregulation of the E-cadherin complex is a frequent process in gastric carcinogenesis. In fact, E-cadherin ectodomain shedding was initially discovered in MCF-7 mammary carcinoma cells [129]. Its oncogenic potential is underlined by the modulation of receptor tyrosine kinases, MAPK and PI3K/Akt/mTOR pathways, which leads to tumor cell growth, survival and motility, and to be strongly elevated in various human tumors [130,131]. Soluble E-cadherin is constantly shed in normal epithelial cells to a low extent, but increased in primary and metastasizing prostate, ovarian, lung or gastric tumors, which correlates with histopathological grade, metastasis recurrence and decreased survival [132,133,134]. Since E-cadherin ectodomain shedding serves as a biomarker for gastric cancer, our recent studies on HtrA-mediated E-cadherin cleavage focused the molecular interaction of HtrA and E-cadherin. HtrA targets the calcium binding sites between the individual repeats in the extracellular domain containing the conserved signature sequence [VITA]-[VITA]-x-x-D-[DN]. Calcium binding, which is required for proper homophilic interaction of the extracellular E-cadherin domains and functional adhesive functions, limits HtrA-mediated E-cadherin cleavage likely through masking the HtrA recognition and cleavage sites [135,136]. To block HtrA activity, several small molecule compounds and a substrate-derived inhibitory peptide were developed that efficiently hindered E-cadherin cleavage in vitro and bacterial transmigration [46,136,137,138,139,140]. These data indicate that the E-cadherin-cleaving properties of *H. pylori*-secreted HtrA can be pharmacologically blocked, serving as potent lead structures for therapeutic interventions in the future.

Generally, the soluble extracellular domain of E-cadherin furthers the disruption of intercellular adhesion between E-cadherin-positive cells in a paracrine loop [141,142], but also leads to a disintegration of the intracellular complex and to the release of β-catenin followed by its nuclear translocation and transactivation of cancer-associated target genes [143]. The extracellular release might be followed by an intracellular cleavage by presenilin-1/γ-secretase [144]. The observation that further cleavage events at the intracellular domains in *H. pylori*-infected epithelial cells [46] implies that HtrA does not simply open intercellular adhesions, but also deregulates intracellular signal transduction initiated by *H. pylori*-triggered E-cadherin shedding. In fact, α-catenin dissociated from E-cadherin, while β-catenin and p120 catenin still colocalized with internalized E-cadherin [145]. Additional studies indicated that β-catenin and p120 catenin localize in the nucleus of *H. pylori*-infected cells, which leads to the transactivation of Tcf/Lef controlled genes, including *mmp-7*, *c-myc*, *cyclin d1*, etc. [9,94,146,147,148,149,150,151]. A functional association between MMP- or HtrA-triggered E-cadherin shedding and catenin signaling needs to be established in future; however, it may represent an effective mechanism in the induction and progression of gastric cancer.

## 4. Concluding Remarks

Regulated protease activity is crucially important in the homeostasis of the gastric epithelium. *H. pylori* actively interferes with host cells through the activation of cellular proteases and the secretion of bacterial proteases, which possibly are involved in the induction and progression of gastric cancer. Since not all proteases are characterized in detail, future work is necessary to examine expression, activity and substrates in vitro and in vivo. In particular, extracellular proteases represent significant targets for novel pharmacological compounds to treat gastric cancer and to prevent metastasis.

## Figures and Tables

**Table 1:** Classification of important MMPs (according to [32]).

MMP Type	Subgroup	Members
Soluble MMPs	*Collagenases*	MMP-1, -8, -13
	*Gelatinases*	MMP-2, -9
	*Stromelysins*	MMP-3, -10, -11
	*Heterogeneous Group*	MMP-7, -12, -26, -28
Membrane anchored MMPs	*MT-MMPs*	MMP-14, -15, -16, -17, -24, -25,

**Table 2:** Proteolysis in *H. pylori*-associated disease.

Protease	Putative Target	Inducing Factors	Involved Signalling Pathways	Cellular Response
ADAM-10	E-cadherin, c-Met, Notch1	*H. pylori* [39]	unknown	loss of AJ [ 47], stem-like phenotype in cancer stem cells [48]
ADAM-17	proTNF-α, TGF-α, HB-EGF	CagL, IL-8, LPS *H. pylori cag*PAI+/− [41]	Rac1, p38, EGFR transactivation via HB-EGF	pro-inflammtory response [ 41,42], reduces apoptosis [44]
MMP-1	vitronectin, fibronectin, collagen, proTNF-α, proIL-1β, proMMP-2, proMMP-9	*cag*PAI, OipA, gastrin	PKC, JNK, Erk, p38	migration through collagen matrices [ 62], activation of gelatinase-type MMPs [58]
MMP-8, -13	unknown	unknown	unknown	unknown
MMP-2, -9	ECM, type IV collagen	MMP-9: CagA * MMP-2: unknown	MMP-9: NF-κB, Erk MMP-2: unknown	neutrophil recruitment [ 67], invasive growth, angiogenesis [32,78]
MMP-3	unknown	pCagA *, IL-1 β	unknown	EMT [ 80], migration and invasion [85]
MMP-10	unknown	CagA *	EGFR, Src, Erk	Invasion [ 51]
MMP-11	unknown	unknown	unknown	IGF-1 production, proliferation, invasion [ 88]
MMP-7	proTNF-α, HB-EGF, E-cadherin, IGFBP5	*cag*PAI, gastrin	NF-κB, MEK, p120	EMT [ 93], migration, invasion [89], IGFII release [97]
MMP-12	unknown	unknown	unknown	macrophage transmigration, reduced angiogenesis [ 32]
Hp0169	Type I collagen	na	unknown	Colonization of Mongolian gerbils [ 114]
HtrA	E-cadherin, fibronectin	na	unknown	Disruption of AJs, access to intercellular space [ 46,125]

* In vitro and in vivo data are inconsistent; na, not applicable.
